# The influence of a plant-based diet on the composition and functions of the human gut microbiota: a review

**DOI:** 10.3389/fnut.2026.1774375

**Published:** 2026-02-17

**Authors:** Erika Egas-Montenegro, Jennifer Echeverria-Chilla, Marcela García-Ulloa, Cristina Aizaga-Benalcazar, Roberto Ordoñez-Araque

**Affiliations:** 1 Facultad de Salud y Bienestar, Escuela de Nutrición y Dietética, Universidad Iberoamericana del Ecuador (UNIB.E), Quito, Ecuador; 2 Grupo de Investigación en Alimentos y Agroindustria (GIA^2^), Escuela de Gastronomía, Universidad de las Américas (UDLA), Quito, Ecuador

**Keywords:** dietary fiber, gut microbiota, metabolic functions, microbial composition, plant-based diet

## Abstract

The human gut microbiota represents a highly diverse ecosystem, with its composition and functions influenced by dietary, biological, and environmental factors. This research aimed to analyze how diet significantly impacts microbial dynamics, particularly through the consumption of dietary fiber, micronutrients, and bioactive compounds. A comprehensive review of major scientific databases was conducted to identify and evaluate studies that explored the relationship between diet and gut microbiota, as well as the primary microbial genera and species linked to these dietary patterns. The primary bacterial phyla present in the human gut include Firmicutes, Bacteroidetes, Actinobacteria, and Verrucomicrobiota. Among these phyla, notable genera include *Bacteroides, Prevotella, Faecalibacterium, Bifidobacterium, and Akkermansia*. These taxa are integral to several essential processes, such as the degradation of complex polysaccharides, the synthesis of short-chain fatty acids, the modulation of immune responses, and the preservation of the intestinal barrier. From a nutritional perspective, current evidence indicates that a plant-based diet may enhance microbial diversity, augment the production of beneficial metabolites such as short-chain fatty acids, and facilitate bacterial profiles that are associated with anti-inflammatory properties, metabolic regulation, and the maintenance of epithelial integrity. In conclusion, dietary patterns that are particularly rich in fiber, micronutrients, and bioactive compounds derived from plant sources have a profound impact on the composition and functions of the gut microbiota. A thorough understanding of these interactions is crucial for developing nutritional strategies that enhance both gut health and overall well-being.

## 1 Introduction

A plant-based diet has been extensively recognized as a beneficial dietary model, demonstrating significant advantages for gut microbiota and playing a crucial role in the prevention and management of various health conditions. These conditions include cardiovascular disease, diabetes mellitus, cancer, chronic kidney disease, obesity, cataracts, and metabolic syndrome, as well as disorders that impact brain and bone health (1, 2). Numerous studies have established a correlation between adherence to this dietary pattern and reduced mortality rates, alongside diminished risks of hypertension (up to 75%), diabetes mellitus (47–78%), and cancer (14%) (1, 3). Furthermore, individuals who adopt a plant-based diet frequently engage in additional health-promoting behaviors. These behaviors may encompass regular physical activity, smoking cessation, reduced or eliminated alcohol consumption, enhanced social interactions, and the implementation of strategies aimed at emotional regulation and cognitive and behavioral improvement. The integration of these biological, psychological, and social components contributes to the amplified health benefits associated with a plant-based dietary approach (4, 5).

Nutrients derived from plant-based foods are essential for the maintenance of a healthy gut microbiota, fostering an optimal environment for a diverse range of beneficial microorganisms. This balanced microbial ecosystem is crucial for the proper functioning of various physiological systems, including the immune, nervous, endocrine, and gastrointestinal systems. Furthermore, plant-based foods are abundant in phytochemicals, such as carotenoids and polyphenols, which are bioactive compounds known for their multiple beneficial effects at the cellular level. These compounds primarily function as antioxidants, effectively neutralizing reactive oxygen and nitrogen species, including free radicals (6, 7). Adopting a plant-based diet promotes optimal health by encouraging the formation of diverse and stable microbial communities. Numerous studies have indicated that individuals who adhere to this dietary pattern exhibit a higher abundance of specific bacterial taxa considered beneficial to human health compared with those following omnivorous diets. A diverse bacterial community is capable of producing a broad spectrum of functional metabolites that exert systemic and metabolic effects on the host, thereby shaping what is recognized as the gut microbiome (8, 9).

Diet quality is a significant determinant of gut microbiota composition, subsequently influencing the overall microbiome configuration. The typical Western diet, characterized by a high intake of animal fats and proteins, elevated sugar consumption, and low fiber intake, is associated with diminished microbial diversity and richness. Conversely, dietary patterns that are low in fat, restrict animal products and sugar, and are high in fiber contribute to the enrichment of intestinal bacterial composition and promote the synthesis of protective metabolites (10). Dietary fiber is a fundamental component of plant-based foods that plays a significant role in health promotion. Current guidelines recommend an intake of 25–32 grams per day for women and 30–35 grams per day for men (11); however, actual consumption levels are frequently below these recommendations. For instance, in Nordic countries, women’s fiber intake ranges between 16 to 22 grams per day, while men’s intake ranges from 18 to 26 grams per day (12). Increasing dietary fiber consumption through fruits, vegetables, legumes, and whole grains is an advantageous public health initiative (13, 14). A growing body of evidence indicates that higher fiber intake is associated with a reduced risk of various noncommunicable diseases, including cardiovascular disease, pancreatic cancer, and diverticular disease. Furthermore, increased fiber consumption is correlated with improvements in lipid profiles, glycemic regulation, and intestinal function. Dietary fiber also fosters a diverse and functional gut microbiota, which enhances the production of short-chain fatty acids that can modulate inflammatory pathways and support metabolic homeostasis. Collectively, these findings underscore the importance of prioritizing fiber-rich foods to mitigate the burden of noncommunicable diseases within populations (15–17).

Diet significantly influences the gut microbiota, affecting its composition, diversity, and metabolic functions, which have profound implications for human health. Various dietary patterns, particularly those emphasizing plant-based foods, have been demonstrated to enhance the abundance of beneficial microorganisms and the production of critical metabolites, such as short-chain fatty acids (18). Among these dietary patterns, vegetarian, vegan, Mediterranean, flexitarian, and whole food plant-based diets exhibit a high intake of dietary fiber, phytochemicals, and unsaturated fats. However, they differ in their degree of exclusion of animal-based foods and flexibility regarding dietary choices. The specific characteristics of these diets promote distinct microbial profiles that are associated with anti-inflammatory effects, improvements in cardiometabolic health, and the strengthening of the intestinal barrier (18, 19). These findings underscore the significance of dietary patterns as a vital strategy for modulating intestinal eubiosis and promoting overall health.

The objective of this research was to examine the relationships between the primary taxonomic groups of the human gut microbiota and plant-based dietary patterns. The aim was to identify the mechanisms through which plant nutrients influence microbial diversity, the production of beneficial metabolites, and the integrity of the intestinal barrier.

## 2 Integration of scientific evidence and results discussion

### 2.1 Composition and functional aspects of the gut microbiota

The gut microbiota is predominantly composed of two major bacterial phyla: Firmicutes and Bacteroidetes, which collectively account for approximately 90% of the microbial community. Additionally, Actinobacteria are present in smaller proportions. The Firmicutes phylum includes notable genera such as *Lactobacillus* and *Clostridium*. In contrast, the Bacteroidetes phylum is primarily represented by the genera Bacteroides and *Prevotella*, while the most significant genus within Actinobacteria is *Bifidobacterium*. The composition and balance of these microorganisms are vital for maintaining homeostasis within the body. Given their functional significance and regulatory roles in various physiological processes, the gut microbiota has been proposed to function as an additional organ within the human body, with an importance that parallels that of other essential biological systems (20, 21).

The composition of the gut microbiota changes based on various factors, including the types of bacteria that are introduced and how they multiply. These changes are influenced by diet, the availability of food substrates, variations in intestinal transit time, luminal pH, host secretions, and the regulation of gene expression in both the individual and their microbiota (22).

#### 2.1.1 Dominant enterotypes

The gut enterotype describes the dominant microbial community structures present in the human gastrointestinal tract, characterized by specific combinations of prevalent bacterial genera. This microbial composition is not static; it undergoes changes throughout an individual’s life span, influenced by various factors including the quality of diet (especially long-term dietary habits) environmental conditions, age, physiological state, and certain pharmacological interventions (23).

In the field of microbiome research, three primary enterotypes have been characterized. The first enterotype, dominated by bacteria of the genus Bacteroides, is predominantly observed in individuals who adhere to diets high in animal proteins and fats. This enterotype is associated with a pro-inflammatory profile and an elevated risk of metabolic syndrome and other cardiometabolic disorders. The second enterotype is characterized by a predominance of Prevotella, which is commonly observed in populations consuming high amounts of complex carbohydrates and dietary fiber. This enterotype has been associated with anti-inflammatory effects and a metabolically protective phenotype. The third enterotype is dominated by Ruminococcus; however, its functional characterization remains less defined and necessitates further scientific investigation to elucidate its biological contributions. Preliminary findings suggest a link to mucin degradation and carbohydrate metabolism (24–26).

**Figure 1:**
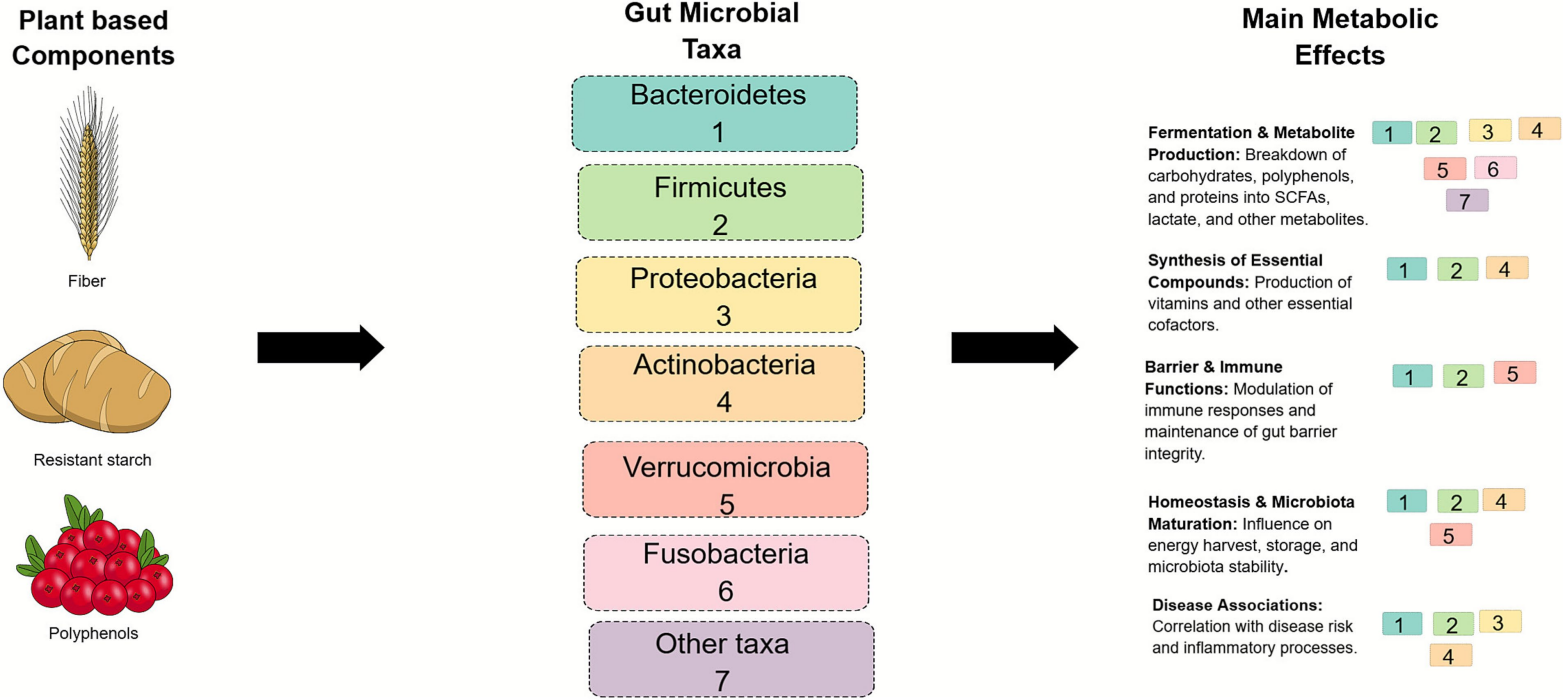
Interaction between plant-based dietary components, microbial taxa, and major metabolic effects. Conceptual schematic illustrating the interactions between key plant-based dietary components, major gut microbial taxa, and their associated metabolic and physiological effects on host health. These effects include microbial fermentation, metabolite production, and modulation of inflammatory and metabolic pathways. Numbers indicate the microbial taxa involved in each process. This figure was created using Mind the Graph (www.mindthegraph.com).

Table 1 provides an overview of the major bacterial phyla and genera of the gut microbiota and their predominant functions, while Figure 1 illustrates the interaction between plant-based dietary components, key microbial taxa, and their main metabolic effects.

**Table 1:** Major taxa of the gut microbiota and their biological functions.

Taxa	Description and functions	Source
Bacteroidetes		
*Bacteroides*	Vitamin-prototrophic bacteria capable of synthesizing several B-complex vitamins; key contributors to carbohydrate fermentation and SCFA production.	(27)
*Prevotella*	Participates in glucose metabolism by enhancing glycogen storage; associated with anti-inflammatory effects in conditions such as inflammatory arthritis and multiple sclerosis.	(1)
*Alistipes*	Exhibits species-specific effects: some strains may promote healthy phenotypes (e.g., protection against colitis and metabolic disorders), while others have been associated with anxiety, depression, ME/CFS, and colorectal cancer.	(28)
*Rikenellaceae*	Produce acetate and propionate; may modulate adiposity and energy homeostasis.	(29)
Proteobacteria		
*Escherichia coli*	Capable of degrading GABA into succinate, which can enter the Krebs cycle; includes both commensal and pathogenic strains.	(30)
*Shigella* spp.	Highly adherent and invasive pathogens that disrupt epithelial homeostasis and rapidly induce intestinal inflammation.	(31)
Firmicutes		
*Clostridium* spp.	Utilize amino acids as energy sources, promoting proteolytic fermentation and generating branched-chain fatty acids, which may contribute to insulin resistance and elevated LDL cholesterol.	(30)
*Faecalibacterium prausnitzii*	A major butyrate producer with essential roles in immune modulation; requires external sources of vitamins (auxotrophic). Produces vitamins K2 and multiple B vitamins.	(30)
*Eubacterium* spp.	Located in the distal colon; ferment soluble fibers into SCFAs such as butyrate, propionate, and lactate.	(30)
*Streptococcus* spp.	Ferment carbohydrates to produce lactic acid; include both commensal and opportunistic species.	(32)
*Roseburia* spp.	Butyrate-producing bacteria associated with improved glucose tolerance and reduced risk of type 2 diabetes.	(30)
*Lactobacillus* spp.	Synthesize GABA and exhibit resistance to glutamic acid; important for mucosal immunity and carbohydrate fermentation.	(30)
*Bacillus* spp.	Capable of synthesizing neurotransmitters such as dopamine and norepinephrine; some strains exhibit probiotic properties.	(30)
*Enterococcus* spp.	Opportunistic pathogens associated with endocarditis, urinary tract infections, and intra-abdominal infections.	(33)
*Ruminococcus* spp.	Degrade dietary fiber and produce SCFAs including butyrate, propionate, and acetate.	(30)
*Ruminococcus bromii*	Key degrader of resistant starch; present in >90% of human fecal samples; essential for SCFA production from starch substrates.	(34)
*Eubacterium rectale*	Major butyrate producer involved in energy homeostasis, colon motility, immune regulation, and inflammation control.	(35)
*Lachnospiraceae*	Produce SCFAs, particularly butyrate; contribute to gut barrier integrity and immune homeostasis.	(30)
Actinobacteria		
*Bifidobacterium* spp.	Produce acetate and contribute indirectly to butyrate formation; essential for intestinal barrier protection, immune development, and colonization resistance.	(36)
Verrucomicrobia		
*Akkermansia* spp.	Improve glucose tolerance and exert protective effects against type 2 diabetes.	(30)
*Akkermansia muciniphila*	Mucin-degrading bacterium representing 3–5% of the microbiota in healthy adults; associated with enhanced gut barrier function, reduced bacterial translocation, improved metabolic health, and protection against obesity.	(1)
Fusobacteria		
*Fusobacterium* spp.	(Corrected) Some species are linked to pathogenic processes, including inflammatory conditions and colorectal cancer, though associations with type 2 diabetes remain inconclusive.	(30)
Other genera of the gut microbiota		
*Bilophila* spp.	Bile-resistant bacteria associated with intra-abdominal abscesses, appendicitis, and hepatic abscesses; linked to inflammatory responses.	(37)
*Veillonella* spp.	Ferment lactate into propionate; may contribute to oral health and exercise metabolism.	(38)
*Blautia* spp.	Support microbiota maturation in children and play a role in recovery from *Vibrio cholerae* infection.	(39)

The table organizes microorganisms according to their taxonomic classification. Each major header corresponds to a bacterial phylum, under which the predominant genera of the human gut microbiota are listed. The functions described refer to the most relevant physiological and metabolic roles attributed to these genera. The category “other gut microbiota genera” includes relevant microorganisms that do not cluster within a single predominant phylum but nevertheless play important roles within the intestinal ecosystem.

#### 2.1.2 Functional aspects of the gut microbiota

The gut microbiota is integral to numerous metabolic and nutritional processes that facilitate digestion. It plays a key role in the fermentation of indigestible components, enhances nutrient absorption, and is involved in the synthesis of various metabolites, referred to as postbiotics, including short-chain fatty acids (SCFAs) and phytoestrogens (40). Moreover, the gut microbiota plays a critical protective role by promoting mucus production in intestinal epithelial cells, thereby forming a barrier against pathogenic microorganisms and environmental toxins. In addition, it supports gastrointestinal maturation, maintains intestinal mucosal integrity, ensures appropriate enzymatic activity, and contributes to host immune defense by regulating local immune responses. This multifaceted role underscores the importance of the gut microbiota in both metabolic function and immune regulation (41, 42).

#### 2.1.3 Postbiotics and their functions in the body

Postbiotics are bacterial metabolites produced or secreted by intestinal microbiota, as well as molecules released during bacterial lysis. These compounds are recognized as bioactive substances with potential health benefits for the host, as they can enhance immunomodulatory, immunostimulatory, neuroregulatory, and antimicrobial functions (43). The category of postbiotics encompasses a diverse array of compounds, including short-chain fatty acids (SCFAs) such as butyrate, acetate, and propionate, as well as phytoestrogens, organic acids, enzymes, extracellular polysaccharides, gamma-aminobutyric acid (GABA), vitamins, and ribosomal peptides, among others (44).

These substances exhibit mechanisms akin to probiotics by having the capacity to modify intestinal pH and suppress the proliferation of pathogenic microorganisms, thereby contributing to intestinal homeostasis. Moreover, postbiotics can exert their effects independently of live microbial cells, enabling direct modulation and stimulation of the host’s immune response (45). As detailed in Table 2, postbiotics possess a range of beneficial properties, including anti-inflammatory, intestinal barrier-strengthening, immunomodulatory, anti-adhesive, antihypertensive, antiproliferative, antiviral, hypocholesterolemic, and antioxidant activities. Collectively, these bacterial metabolites are regarded as crucial factors in promoting health due to their ability to modulate physiological processes and support the prevention or alleviation of various pathologies (46).

**Table 2:** Major postbiotics produced by the gut microbiota, associated microorganisms, and their biological functions.

Postbiotic metabolite	Microbial taxa associated with production	Biological functions	Source
GABA	*Lactobacillus* spp.; *Bifidobacterium* spp. (via glutamate decarboxylation)	Enhances visual function; cardioprotective via attenuation of inflammatory responses and modulation of ischemic metabolic pathways; antihypertensive, anxiolytic and antidepressant effects; supports hepatic function and immune responsiveness; inhibitory neurotransmission in the CNS; antitumor activity through suppression of proliferation and induction of apoptosis; renoprotective effects; promotes dermal fibroblast density and cell-volume homeostasis; modulates growth hormone secretion.	(47)
SCFAs	Produced by bacterial fermentation of dietary fiber. Firmicutes (e.g., *Faecalibacterium prausnitzii*, *Roseburia* spp.): butyrate Bacteroidetes (*Bacteroides* spp.): propionate Actinobacteria (*Bifidobacterium* spp.): acetate, lactate Verrucomicrobia (*Akkermansia muciniphila*): propionate	Major metabolites of microbial fermentation that modulate host metabolism and immunity: acetate—most abundant, substrate for peripheral tissues, supports lipid/glucose homeostasis and barrier integrity; propionate—hepatic metabolism lowers serum cholesterol and glucose, regulates adipose stores and exerts anti-inflammatory/cardiometabolic effects; butyrate—primary energy source for colonocytes, promotes epithelial repair, immune cell differentiation, anti-inflammatory and antitumor actions; collectively regulate gene expression, preserve intestinal and blood–brain barriers.	(48)
Teichoic acids	*Lacticaseibacillus paracasei* D3-5 *Lactobacillus delbrueckii* *Latilactobacillus sakei* *Lacticaseibacillus rhamnosus* GG *Lactiplantibacillus plantarum* K8	Determine bacterial cell morphology and division; modulate metabolic aspects of bacterial physiology; inhibit proinflammatory cytokine expression (e.g., IL-8) at the intestinal epithelium; disrupt biofilm formation and stability; ameliorate high-fat–diet–induced metabolic dysfunctions by reducing intestinal permeability and inflammation; balance Th1/Th2 responses and exert immunomodulatory activity.	(49)
Bacteriocins	Lactic acid bacteria (LAB) Gram-positive and Gram-negative bacteria *Lactobacillus acidophilus* ATCC 4356	Targeted antimicrobial agents with species-specific bacteriostatic/bactericidal activity that spare non-target microbiota; active against foodborne pathogens including *C. difficile* and certain antibiotic-resistant strains; clinically relevant for urogenital and resistant infections; function as food bio-preservatives (notably in dairy); inhibit biofilm formation, adhesion and virulence factor expression.	(50)
CFS	*Lactobacillus* spp. *Lactobacillus acidophilus* *Lactobacillus casei* *Lacticaseibacillus rhamnosus* GG *Lactiplantibacillus plantarum* *Bifidobacterium* spp. *Saccharomyces cerevisiae* *Saccharomyces boulardii*	Antibacterial activity preventing enterocyte invasion; antioxidant properties that accelerate wound healing and barrier restoration; maintain peristalsis under stress and act as anti-infectives in diarrheal disease; modulate inflammation (↓proinflammatory cytokines, ↑IL-10) in epithelial cells and phagocytes; enhance epithelial integrity and cytoprotective gene expression; inhibit invasion/proliferation of colorectal cancer cells and reduce oxidative stress.	(51)
Enzymes (microbial)	Bacteroidetes Firmicutes *Lactobacillus* spp. *Lactobacillus fermentum* *Lactiplantibacillus plantarum*	Provide antioxidant and anti-inflammatory effects; facilitate digestion and nutrient absorption via degradation of complex substrates; produce ROS-detoxifying enzymes (e.g., glutathione peroxidase, SOD, catalase); confer enzymatic activities absent in humans for polysaccharide/polyphenol breakdown and vitamin biosynthesis; contribute to colorectal cancer risk reduction through metabolic modulation.	(52)
Phytoestrogens (microbial metabolites)	Actinobacteria (*Bifidobacterium* spp.) Firmicutes (*Lactobacillus* spp., *Clostridium* spp.) Bacteroidetes (*Bacteroides* spp.)	Exert estrogenic, antioxidant, antiangiogenic and antiproliferative effects; modulate androgen receptor pathways and inhibit tumor-related enzymes (prostate cancer relevance); support bone metabolism; improve lipid profiles and regulate serum cholesterol; inhibit platelet aggregation and thrombin formation in atherosclerotic plaques; reduce vasomotor climacteric symptoms.	(53)
Cell wall fragments	Gram-positive bacteria (*Lactobacillus* spp., *Bifidobacterium* spp.) Gram-negative bacteria (*Bacteroides* spp., *Akkermansia* spp.)	Elicit immunogenic and immunostimulatory responses; stimulate mast cell–mediated defenses in skin; modulate cytokine balance by reducing IL-12 and increasing IL-10; display anti-inflammatory and anticancer activities through immune regulation.	(51)
Bacterial lysates	Gram-positive bacteria (*Lactobacillus* spp., *Bifidobacterium* spp.) Gram-negative bacteria (*Escherichia coli*, *Bacteroides* spp.)	Alleviate atopic dermatitis symptoms; modulate the gut–lung axis via lymphocyte trafficking and enhanced mucosal IgA secretion; reduce incidence and severity of respiratory infections; decrease asthma exacerbations in children and support COPD management in adults; lower frequency of allergic rhinitis episodes.	(51)
Vitamins	Actinobacteria (*Bifidobacterium* spp.) Firmicutes (*Lactobacillus* spp., *Enterococcus* spp., *Clostridium* spp.) Bacteroidetes (*Bacteroides* spp.)	Microbial biosynthesis of biotin, cobalamin, folates, nicotinic acid, pantothenic acid, pyridoxine, riboflavin and thiamine; contribute to neuronal health, DNA synthesis and prevention of megaloblastic anemia through B12/folate production (strain-dependent, e.g., *L. acidophilus*); synthesize vitamin K required for coagulation factor production.	(52)

GABA, Gamma-aminobutyric acid; SCFAs, Short-chain fatty acids (butyrate, acetate, propionate); CFS, Cell-free supernatants. Postbiotics include microbial metabolites and structural components produced by gut microorganisms. Cell-free supernatants contain soluble bioactive molecules free of viable cells. Enzymes are included as postbiotics when produced by gut microbes and exert extracellular activity. Phytoestrogens are classified as microbial metabolites because they result from gut-microbial biotransformation of dietary precursors. Microbial producers are listed at different taxonomic levels (phylum, genus, species, or strain) depending on the available evidence.

### 2.2 Modulatory effects of plant-based diets on the human gut microbiota

The integrity of intestinal barrier function has long been recognized as being closely linked to gut microbiota composition, which is shaped by a range of host and environmental factors. Early-life determinants such as gestational age, mode of delivery, infant feeding practices, and weaning, together with age, ethnicity, lifestyle, geographic context, antibiotic exposure, metabolic disorders, and intestinal comorbidities, have been identified as key modulators of microbial structure and function (54). Among these factors, long-term dietary habits represent one of the most influential determinants of microbial diversity and intestinal homeostasis. Classical evidence indicates that dietary patterns characterized by low fiber intake and high consumption of animal proteins and fats promote persistent alterations in gut microbial diversity, which are associated with increased intestinal permeability and heightened inflammatory responses. These effects are largely mediated by disruptions in bacterial translocation and changes in the production of microbial metabolites involved in immune modulation (55).

More recent mechanistic studies have expanded this framework by identifying specific microbiota-derived metabolites that link diet to cardiometabolic risk, notably trimethylamine N oxide (TMAO). Trimethylamine (TMA), the precursor of TMAO, is produced by gut microbiota during the metabolism of dietary choline and carnitine, nutrients predominantly derived from animal-based foods. Following absorption, TMA is oxidized in the liver to form TMAO, elevated levels of which have been associated with increased cardiovascular risk. Evidence indicates that adherence to plant-based dietary patterns is associated with lower TMAO production, driven both by reduced intake of TMA precursors and by diet-induced shifts in gut microbial composition, including changes in the relative abundance of Firmicutes and Proteobacteria. These phyla encompass taxa with diverse functional capacities, and their contribution to TMAO production depends on metabolic activity and dietary context, ultimately resulting in a reduced overall microbial capacity for TMA formation, even when choline-containing foods are occasionally consumed (56–60).

Building on these findings, recent studies demonstrate that plant-based diets promote the development of a more diverse and functionally resilient gut microbiota compared with omnivorous dietary patterns. Regular consumption of whole grains, fruits, and vegetables is associated with increased microbial diversity and enrichment of taxa within the phylum Bacteroidetes, as well as genera with protective metabolic roles, including Prevotella and Ruminococcus (61). This enhanced diversity is linked to favorable postbiotic production and epigenetic modifications that contribute to reduced risk of chronic inflammation and degenerative diseases (62).

Consistent with this framework, gut microbial diversity has been strongly associated with body mass index (BMI). Overweight and obese individuals typically exhibit a reduced Bacteroidetes: Firmicutes ratio, alongside increased abundance of Proteobacteria and elevated levels of C-reactive protein, reflecting a pro-inflammatory state. In contrast, dietary patterns rich in plant-based foods are correlated with lower body weight and healthier BMI profiles, effects that appear to be mediated by enhanced microbial diversity, improved metabolic balance, and reduced systemic inflammation (63, 64).

#### 2.2.1 Predominant bacterial taxa in plant-based dietary patterns

The bacterial diversity within the gut microbiome is effectively characterized by the *Prevotella: Bacteroides* ratio, which serves as a sensitive indicator of dietary patterns. Empirical research demonstrates that individuals whose diets are abundant in dietary fiber, complex carbohydrates, and resistant starch tend to exhibit a higher prevalence of *Prevotella*. This microbial profile is associated with beneficial metabolic functions. In contrast, individuals who consume diets rich in animal protein and saturated fat typically show a predominance of *Bacteroides*, which correlates with diminished microbial diversity and less favorable metabolic profiles (1, 65).

In this context, Plant-based diets are associated with the enhancement of various beneficial gut microbiota, including genera such as *Lactobacillus*, *Ruminococcus*, *Eubacterium rectale*, *Roseburia*, *Bifidobacterium*, *Veillonella*, *Lachnospira*, *Rikenellaceae*, as well as members of the phylum Verrucomicrobia. These microorganisms play critical roles in the degradation of dietary fiber, the production of postbiotic metabolites, particularly short-chain fatty acids, and the modulation of immune responses. Consequently, they contribute significantly to the prevention and management of cardiovascular, metabolic, immune, and neurodegenerative diseases (30, 66).

#### 2.2.2 Impact of dietary patterns on key microbial taxa

Early studies established that diet is a major determinant of gut microbial structure, giving rise to predominant enterotypes characterized by *Prevotella*, *Bacteroides*, and *Ruminococcus*. Comparative analyses of fecal microbiota across mammalian species and herbivores provided foundational evidence demonstrating that dietary patterns exert a strong influence on intestinal microbial composition. In these studies, the dietary habits of omnivorous mammals closely resembled those of humans consuming mixed diets, whereas herbivorous feeding patterns showed similarities to plant-based dietary regimens in humans (67). These observations laid the groundwork for understanding how diet quality and composition modulate enterotype distribution.

Subsequent research has shown that increased consumption of animal proteins and fats, particularly when combined with long-term inadequate fiber intake, favors the proliferation of *Bacteroides*. This genus exhibits high tolerance to bile salts, conferring a competitive advantage under high-fat dietary conditions and concomitantly reducing the abundance of microorganisms specialized in fermenting complex carbohydrates. In contrast, regular and sustained consumption of plant-based foods, including vegetables, fruits, whole grains, and fiber-rich legumes, has been consistently associated with increased abundance, diversity, and prevalence of *Prevotella* and *Ruminococcus*. These taxa play a key role in polysaccharide degradation and the production of metabolites that support host metabolic and intestinal health (68).

More recent dietary intervention studies have further refined these associations by identifying specific microbial responses to distinct carbohydrate sources. Diets enriched in non-digestible carbohydrates derived from whole grains and wheat bran have been linked to increased abundance of *Bifidobacterium* and *Lactobacillus*, genera characterized by predominantly saccharolytic metabolism and protective effects on intestinal barrier integrity through inhibition of pathogenic bacteria. Additionally, intake of resistant starch, such as that present in whole barley, promotes the growth of lactic acid bacteria including *Eubacterium rectale* and *Roseburia*. Conversely, other members of the phylum Firmicutes, such as *Clostridium* and *Enterococcus*, tend to be less prevalent in gut microbiota shaped by plant-based dietary patterns (1).

##### 2.2.2.1 Modulation of core phyla ratios (bacteroidetes/firmicutes)

Traditionally, the gut microbiota of healthy adults has been described as being characterized by a predominance of *Bacteroidetes* relative to *Firmicutes*, and early research identified alterations in this ratio as a key factor associated with energy harvest and obesity. Classic studies established that a lower *Bacteroidetes* to *Firmicutes* ratio is frequently observed in individuals with overweight and obesity, suggesting a link between microbial composition, caloric extraction, and host metabolic status (69, 70).

Subsequent research demonstrated that dietary patterns play a central role in modulating the relative abundances of these two bacterial phyla, particularly when comparing omnivorous and plant-based diets (71, 72). In this context, Jain et al. (73) examined two child populations with contrasting dietary habits: one from India, characterized by a predominantly plant-based diet, and another from China, with higher consumption of animal-based foods. The authors reported that the proportion of *Bacteroidetes* in the Indian population was approximately four times higher than that observed in the Chinese population, indicating a strong association between reduced intake of animal-derived foods and increased abundance of *Bacteroidetes*. Similarly, De Filippo et al. (74) compared the gut microbiota of Italian children consuming a Western diet rich in animal proteins, fats, sugars, and low in fiber, with that of children from a rural African community whose diet was high in plant-based foods, fiber, and resistant starch. Their findings revealed that *Firmicutes* were present at approximately twice the abundance in Italian children compared to African children, further supporting the role of plant-rich diets in shaping a microbial profile characterized by higher *Bacteroidetes* and lower *Firmicutes* abundance.

Building on these classic observations, more mechanistic studies have proposed functional explanations for the relationship between the *Bacteroidetes* to *Firmicutes* ratio and host energy balance. Diets rich in resistant starch have been shown to reduce the abundance of *Firmicutes* while increasing *Bacteroidetes* and *Bifidobacterium*, contributing to improved metabolic outcomes and obesity prevention. Current evidence suggests that a 20% increase in *Firmicutes*, accompanied by a proportional decrease in *Bacteroidetes*, may be associated with an estimated increase of approximately 150 kcal in daily energy harvest, potentially facilitating long-term weight gain (75). Conversely, increasing the *Bacteroidetes* to *Firmicutes* ratio through high intake of dietary fiber may reduce caloric extraction from the diet and support weight loss (76).

##### 2.2.2.2 Enrichment of beneficial genera

Beyond phylum-level changes, dietary patterns also influence microbial composition at the genus level. Individuals adhering to plant-based diets typically exhibit a greater abundance of *Prevotella*, whereas *Bacteroides* species are more prevalent among individuals consuming omnivorous diets. Although *Bacteroides* plays a commensal role in polysaccharide degradation and host energy metabolism, certain species may act as opportunistic pathogens under dysbiotic conditions. In contrast, *Prevotella* has been associated with anti-inflammatory properties and potential protective effects in conditions such as inflammatory arthritis and multiple sclerosis. Collectively, these findings indicate that plant-rich dietary patterns promote microbial configurations linked to favorable metabolic and inflammatory profiles (1, 77).

Diets that encourage the consumption of non-digestible carbohydrates, such as whole grains and wheat bran, are associated with an increased abundance of beneficial bacteria, particularly *Bifidobacterium* and *Lactobacillus*. These genera, characterized by their saccharolytic metabolism, play a crucial role in maintaining the integrity of the intestinal barrier by inhibiting the colonization and proliferation of pathogenic bacteria. Furthermore, the intake of resistant starch and whole barley is linked to an enhancement of lactic acid bacteria, including *Eubacterium rectale* and *Roseburia* (78, 79).

##### 2.2.2.3 Suppression of opportunistic pathogens

Conversely, other members of the *Firmicutes* phylum, such as *Clostridium* and *Enterococcus*, are typically found in lower abundances in microbiota influenced predominantly by diets rich in plant-based foods (78, 79). This selective suppression of potentially pathogenic taxa contributes to improved intestinal barrier function and reduced inflammatory signaling.

Dietary modifications can lead to rapid changes in the composition of the gut microbiota and in the production of bacterial metabolites, with measurable alterations often identifiable within approximately 1 week of dietary intervention. However, these initial effects are typically modest and reversible, whereas more stable and profound changes are observed with long-term adherence to specific dietary patterns (80). For example, individuals maintaining a vegetarian or vegan diet for a minimum of 3 months demonstrate significant and enduring shifts in microbial structure and microbiome-related immune parameters compared to those adhering to these diets for shorter periods. Consequently, the magnitude and stability of microbial benefits increase proportionally with the duration of adherence to plant-based dietary patterns. Nonetheless, the long-term effects of sustained plant-based diets over several years remain insufficiently characterized and warrant further investigation (67).

##### 2.2.2.4 Dietary modulation of additional beneficial and opportunistic gut bacteria

Diets that encourage the consumption of non-digestible carbohydrates, such as whole grains and wheat bran, are associated with an increased abundance of beneficial bacteria, particularly *Bifidobacterium* and *Lactobacillus*. These genera, characterized by their saccharolytic metabolism, play a crucial role in maintaining the integrity of the intestinal barrier by inhibiting the colonization and proliferation of pathogenic bacteria. Furthermore, the intake of resistant starch and whole barley is linked to an enhancement of lactic acid bacteria, including *Eubacterium rectale* and *Roseburia*. Conversely, other members of the Firmicutes phylum, such as *Clostridium* and *Enterococcus*, are typically found in lower abundances in microbiota influenced predominantly by diets rich in plant-based foods (78, 79).

Dietary modifications can lead to rapid changes in the composition of the gut microbiota and in the production of bacterial metabolites, with measurable alterations often identifiable within approximately 1 week of dietary intervention. However, these initial effects are typically modest and reversible, whereas more stable and profound changes are observed with long-term adherence to specific dietary patterns (80). For example, individuals maintaining a vegetarian or vegan diet for a minimum of 3 months demonstrate significant and enduring shifts in microbial structure and microbiome-related immune parameters compared to those adhering to these diets for shorter periods. Consequently, it can be concluded that the magnitude and stability of microbial benefits increase proportionally with the duration of the dietary pattern. Nonetheless, the effects, scope, and potential benefits of prolonged adherence to plant-based diets over several years have yet to be thoroughly characterized in the literature (67).

### 2.3 Effects of plant-based nutrients and bioactive compounds on gut microbial composition

#### 2.3.1 Nutrient bioavailability and the gut microbiota

Low nutritional bioavailability is a characteristic feature of foods that maintain intact cell walls, possess large particulate structures, and/or have not undergone thermal or mechanical treatments that enhance nutrient release, as is often the case with various raw plant-based foods. The consumption of products with lower bioavailability, such as fresh and minimally processed vegetables, yields systemic benefits. This is attributed to a greater proportion of their constituents reaching the distal regions of the gastrointestinal tract in an intact form, where they function as fermentable substrates for gut microbiota. This process promotes the production of beneficial bacterial metabolites and supports the maintenance of a diverse and stable microbial community. Conversely, a diet rich in ultra-processed foods significantly diminishes the availability of nutrients to the intestinal ecosystem, potentially altering the composition, metabolism, and functionality of the microbiota (8, 81). The composition of gut microbiota plays a crucial role in host health, with dietary nutritional characteristics significantly influencing the regulation, maintenance, and proliferation of beneficial microorganisms. A diet abundant in plant-based foods enhances the microbial synthesis of vitamins and promotes the production of neurotransmitters such as GABA, dopamine, and norepinephrine. Furthermore, it facilitates the generation of metabolites involved in energy homeostasis, intestinal motility, and immunomodulation. This dietary pattern also contributes to the integrity of the intestinal barrier, thereby reducing permeability associated with inflammatory processes, inadequate nutrient absorption, and gastrointestinal disorders, such as Crohn’s disease. Consequently, a plant-based diet is linked to a reduced incidence of various diseases, including cardiovascular, neurodegenerative, immunological, endocrine, and metabolic disorders, including diabetes mellitus (82).

#### 2.3.2 Macronutrients

##### 2.3.2.1 Carbohydrates

Non-digestible carbohydrates, including dietary fiber and resistant starch, as well as carbohydrates with low nutritional bioavailability, reach the colon intact. In this environment, they undergo fermentation by the gut microbiota, which contributes significantly to energy production and the generation of various bacterial metabolites. Additionally, even simple carbohydrates, which are more readily digested, can influence the composition and activity of the microbiota by serving as an additional nutrient source for bacterial communities. This process promotes the synthesis of postbiotics (83, 84). The intake of non-digestible carbohydrates has been associated with an increase in beneficial bacterial populations, such as lactic acid bacteria, *Ruminococcus*, *Eubacterium rectale*, and *Roseburia*, while concurrently reducing the abundance of potentially harmful species, including *Clostridium* and *Enterococcus* (85).

Dietary fiber encompasses a variety of compounds, including inulin, galacto-oligosaccharides (both *α* and *β*), xylo-oligosaccharides, and beta-glucans, all of which exert a prebiotic effect by serving as fermentable substrates for intestinal microbiota. Beta-glucans have been shown to promote the proliferation of beneficial bacterial species such as *Prevotella* and *Roseburia*, leading to an increased production of short-chain fatty acids (SCFAs) (86). Likewise, inulin and oligosaccharides facilitate the growth of bifidobacteria and enhance the synthesis of SCFAs. However, research indicates that individuals following vegan and vegetarian diets may exhibit a lower abundance of bifidobacteria. A potential explanation for this observation is the heightened presence of competing genera, such as *Prevotella*, which may more effectively utilize fiber as an energy substrate, consequently displacing other bacterial populations (85).

Regular consumption of non-digestible carbohydrates offers significant health benefits. In addition to their prebiotic effects, these carbohydrates have been shown to reduce the production of pro-inflammatory cytokines and to lower serum concentrations of triglycerides, total cholesterol, and low-density lipoproteins. Furthermore, they are associated with protective effects against cardiovascular disease and various central nervous system disorders (87).

##### 2.3.2.2 Proteins

Adequate protein intake has been associated with enhanced intestinal microbial diversity, as proteins provide nitrogenous substrates that support microbial growth and metabolic activity. However, it is important to note that animal proteins and plant proteins exert distinct effects on the composition and functionality of the microbiota, largely due to differences in accompanying nutrients such as fiber, fat content, and bioactive compounds (67, 88).

In individuals adhering to omnivorous diets, the frequent intake of animal proteins that are typically accompanied by higher saturated fat levels correlates with a reduced abundance of beneficial bacteria. Specifically, key taxa such as *Roseburia*, *Eubacterium rectale*, and *Ruminococcus bromii*, which are critical for the metabolism of polysaccharides and the production of butyrate, are significantly less prevalent. Conversely, omnivorous diets tend to favor the predominance of bile-tolerant bacteria, including *Bacteroides* and various species of *Clostridia*. This shift in gut microbiota composition may have implications for metabolic health and the fermentation of dietary fibers (89).

High-protein diets, particularly those that primarily source protein from animal origins while simultaneously limiting carbohydrate intake, have been shown to decrease the abundance of butyrate-producing bacteria. This reduction is associated with elevated levels of pro-inflammatory markers and an increased risk of developing colorectal cancer. In contrast, diets that emphasize plant-based proteins, such as pea protein, facilitate the growth of beneficial gut bacteria, including *Bifidobacterium* and *Lactobacillus*. These diets also contribute to the suppression of potentially pathogenic microorganisms, such as *Bacteroides fragilis* and *Clostridium perfringens* (90).

##### 2.3.2.3 Fats

The types and quantities of dietary fats consumed play a crucial role in shaping the composition of the gut microbiota. Plant-based diets are generally characterized by lower levels of total and saturated fats, which are conducive to the proliferation of *Bifidobacterium*. Furthermore, vegetable fats are predominantly composed of monounsaturated and polyunsaturated fatty acids. These fatty acids have been associated with an increased ratio of Bacteroidetes to Firmicutes and a greater abundance of beneficial microbial populations, including lactic acid bacteria, *Bifidobacterium*, and *Akkermansia muciniphila* (2, 91). Conversely, a high intake of saturated fats, predominantly sourced from meat products and ultra-processed foods, is associated with an increased abundance of *Bilophila* and *Faecalibacterium prausnitzii*, while simultaneously reducing the concentrations of *Bifidobacterium*. This alteration in the gut microbiome can induce proinflammatory responses by enhancing the production of cytokines, including IL-1, IL-6, and TNF-*α*, thereby facilitating the onset of metabolic disorders. Furthermore, both saturated and trans fats are linked to a decrease in the prevalence of beneficial microbial groups such as Bacteroidetes, *Prevotella*, *Lactobacillus* spp., and *Bifidobacterium* spp., while promoting a relative increase in Firmicutes (92, 93).

#### 2.3.3 Micronutrients

##### 2.3.3.1 Vitamins

The adequacy of vitamin levels in the human body is, in part, contingent upon a diverse and functional gut microbiota capable of performing essential metabolic and biosynthetic processes. Certain vitamins, including vitamin K and various B vitamins (such as riboflavin and folate), can be synthesized by specific bacterial populations residing in the gastrointestinal tract. For instance, *Bifidobacterium* is implicated in the synthesis of vitamin K, biotin, folate, thiamine, and, to a lesser extent, cyanocobalamin. Additionally, *Bacillus subtilis* and *Escherichia coli* are responsible for producing riboflavin, while members of the Lactobacillus genus can synthesize cobalamin and other B vitamins. Available scientific literature indicates that individuals adhering to a plant-based diet exhibit a heightened microbial capacity for folate biosynthesis in comparison to those following an omnivorous diet (94, 95).

##### 2.3.3.2 Minerals

Minerals, along with vitamins and other trace elements, are crucial for both physiological functions in the body and the maintenance of gut microbiota. These minerals operate as cofactors in numerous metabolic pathways and are fundamental to the effective functioning of the immune system. The efficiency of the immune response is significantly influenced by intestinal integrity, as well as the diversity and stability of the resident microbial ecosystem, accounting for approximately 70–80% of immune system effectiveness. Essential minerals, including copper, iron, zinc, selenium, and magnesium, possess immunomodulatory properties that can affect the host’s susceptibility to various diseases and infections. In addition to their role in immunology, these micronutrients are integral to microbial metabolism and serve as vital resources for the various bacterial strains that comprise the gut microbiota (96, 97).

#### 2.3.4 Phytochemicals

Research has demonstrated that only 5–10% of dietary phytochemicals are absorbed in the small intestine, while approximately 90–95% are transported to the colon, where they undergo transformation by the bacterial communities within the microbiota (19). These microbial transformations yield metabolites with significant biological activity, which may exert beneficial systemic effects on the host, including cardioprotective properties and modulation of glucose metabolism. The regular consumption of plant-based foods increases the availability of polyphenols, compounds that are associated with the prevention of metabolic disorders such as obesity and type 2 diabetes mellitus, both of which are characteristic of metabolic syndrome. Furthermore, a variety of phytochemicals have the capacity to modulate gut microbial composition. For example, phenolic compounds present in tea have been shown to inhibit the growth and adhesion of *Clostridium* spp., *E. coli*, and *Salmonella typhimurium* (98, 99).

Flavonoids have been linked to favorable alterations in the Bacteroidetes to Firmicutes ratio, as well as a reduction in Proteobacteria at the phylum level. These changes may confer benefits for individuals with metabolic syndrome and enhance fecal microbial diversity. Furthermore, flavonoids have been shown to impact glucose metabolism and amino acid fermentation, potentially offering protective effects against obesity. Increased serum levels of carotenoids are associated with a reduced risk of chronic diseases and a microbiome that is healthier from both functional and metabolic perspectives. Similarly, glucosinolates present in cruciferous vegetables may facilitate the inoculation and reinoculation of specific bacterial populations, promoting a rise in the proportion of Bacteroidetes by up to 8 percent according to some studies in relation to Firmicutes (100, 101).

Finally, allicin, a prominent phytochemical found in garlic, exhibits significant anti-inflammatory properties and plays an important role in regulating cardiovascular health indicators, including blood pressure, arterial stiffness, and vascular function. Additionally, garlic consumption has been associated with an increased abundance of beneficial gut microbiota, specifically *Lactobacillus* and *Clostridium* spp., which are recognized for their positive effects on metabolic and cardiovascular health (102).

**Figure 2:**
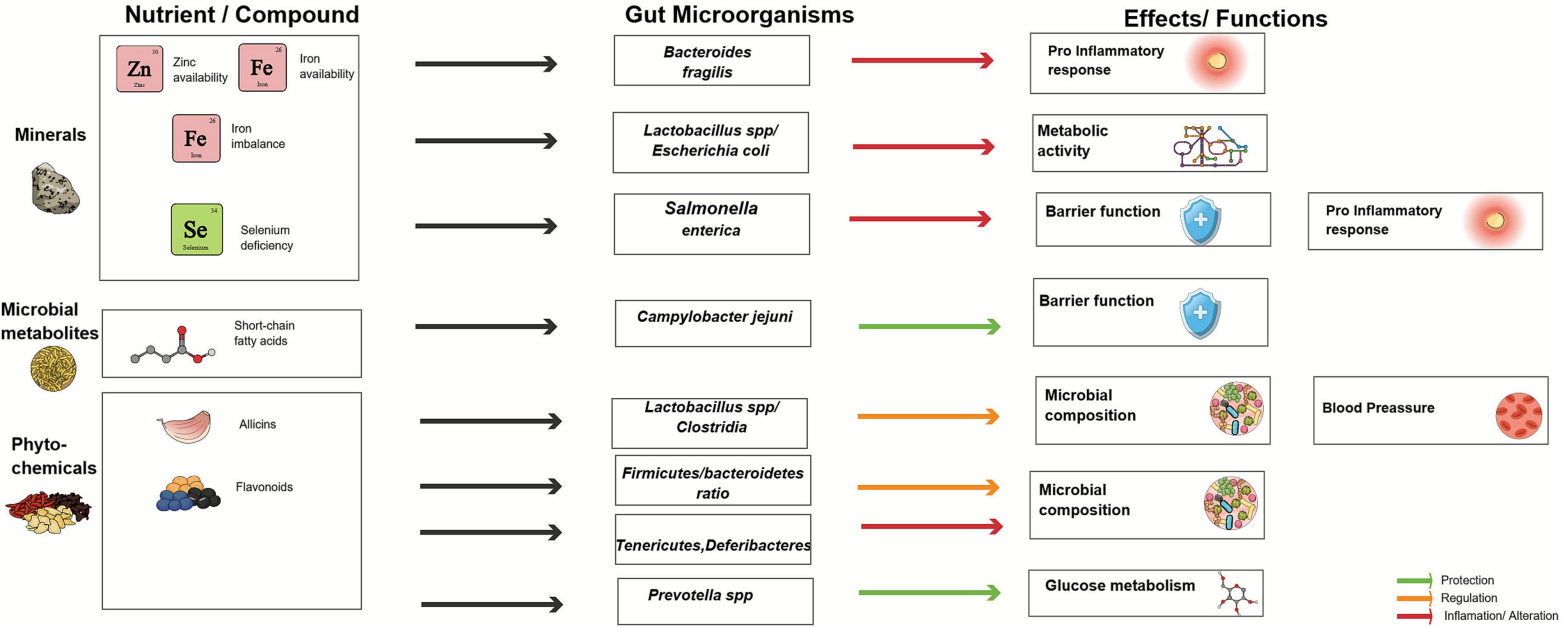
Effects of minerals, microbial metabolites, and phytochemicals on gut microbiota. Conceptual schematic showing the interactions between minerals, short-chain fatty acids, and phytochemicals with gut microbiota. These interactions modulate the abundance and activity of key microbial taxa, influencing inflammation, intestinal barrier function, microbiota composition, and metabolic outcomes. Colored arrows indicate protective, regulatory, or pro-inflammatory/altering effects. This figure was created using Mind the Graph (www.mindthegraph.com).

**Figure 3:**
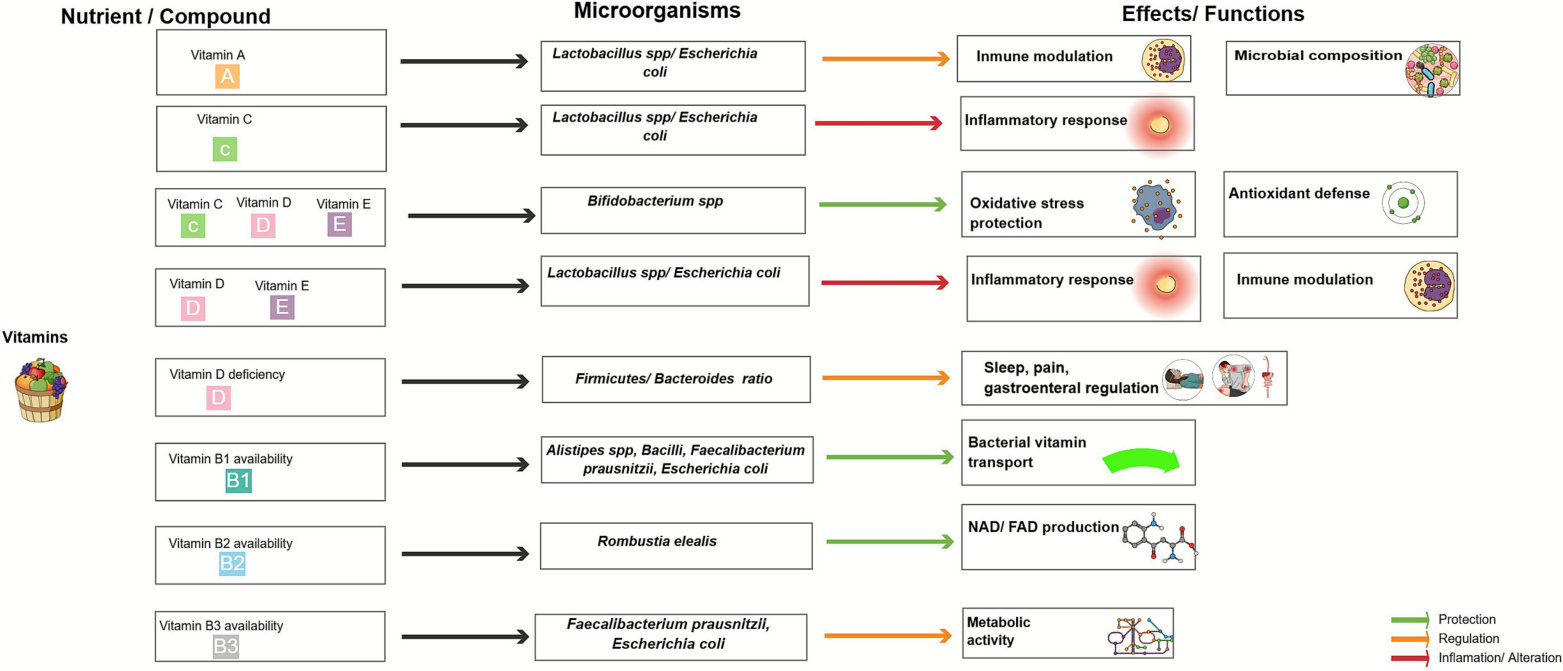
Effects of vitamins on gut microbiota. Conceptual schematic illustrating interactions between vitamins and gut microbiota. These interactions influence pro-inflammatory responses, oxidative stress, gastrointestinal functions, microbial composition, and metabolite production. Colored arrows indicate protective, regulatory, or pro-inflammatory/altering effects. This figure was created using Mind the Graph (www.mindthegraph.com).

Table 3 presents a comprehensive overview of the effects of various micronutrients (including vitamins and minerals) and phytochemicals on gut microbiota. It outlines their influence on beneficial microorganisms, such as *Bifidobacterium*, *Prevotella*, *Lactobacillus*, and *Faecalibacterium prausnitzii*, alongside potentially pathogenic bacteria, including *Bacteroides fragilis*, *Salmonella Typhimurium*, and *Campylobacter jejuni*. These relationships are further illustrated in Figures 2, 3, which provide a schematic representation of the interactions between micronutrients, phytochemicals, and gut microbial taxa. This synthesis underscores the role of micronutrients and bioactive compounds derived from plant-based foods in modulating key physiological processes, including inflammation, oxidative stress, intestinal barrier integrity, and microbial composition. These factors collectively contribute to the maintenance of host health.

**Table 3:** Effects of micronutrients and phytochemicals on the gut microbiota.

Nutrient/Compound	Microorganisms	Effect/Function
Selenium	*Salmonella enterica* serovar Typhimurium	Selenium deficiency impairs intestinal barrier function and promotes inflammatory responses during *S. typhimurium* infection.
Zinc	*Bacteroides fragilis*	Zinc deficiency enhances the pro-inflammatory activity associated with *B. fragilis*.
Iron	*Bacteroides fragilis*	Altered iron availability modulates inflammatory responses linked to *B. fragilis*.
	*Lactobacillus* spp., *Escherichia coli*	Iron imbalance affects the growth and metabolic activity of commensal *Lactobacillus* and *E. coli*.
SCFAs	*Campylobacter jejuni*	SCFAs modulate epithelial barrier integrity and influence the survival of *C. jejuni* in the gut environment.
Vitamin A	*Lactobacillus* spp., *Escherichia coli*	Vitamin A levels influence mucosal immune responses that regulate *Lactobacillus* and *E. coli* populations.
Vitamin B1 (Thiamine)	*Alistipes* spp., *Bacilli*, *Faecalibacterium prausnitzii*, *Escherichia coli*	Thiamine availability affects bacterial thiamine transport and supports survival under low-oxygen conditions.
Vitamin B2 (Riboflavin)	*Romboutsia ilealis*	Riboflavin supports *de novo* synthesis of purines and pyrimidines and contributes to NAD/FAD production in *R. ilealis*.
Vitamin B3 (Niacin)	*Faecalibacterium prausnitzii*, *Escherichia coli*	Niacin promotes bacterial tolerance to low-oxygen environments and modulates metabolic activity.
Vitamin C	*Lactobacillus* spp., *Escherichia coli*	Vitamin C status influences inflammatory responses and mucosal immunity affecting these taxa.
	*Bifidobacterium* spp.	Vitamin C contributes to oxidative-stress protection in *Bifidobacterium*.
Vitamin D	*Bacteroides fragilis*	Vitamin D deficiency is associated with enhanced inflammatory responses linked to *B. fragilis*.
	*Lactobacillus* spp., *Escherichia coli*	Vitamin D modulates mucosal immunity affecting *Lactobacillus* and *E. coli*.
	*Bifidobacterium* spp.	Vitamin D supports antioxidant defense mechanisms in *Bifidobacterium*.
	Firmicutes/Bacteroidetes ratio	Vitamin D status influences the Firmicutes/Bacteroidetes ratio and is linked to sleep regulation, pain, and gastrointestinal conditions.
Vitamin E	*Lactobacillus* spp., *Escherichia coli*	Vitamin E availability modulates inflammatory responses and mucosal immunity affecting these taxa.
	*Bifidobacterium* spp.	Vitamin E contributes to oxidative-stress protection in *Bifidobacterium*.
Flavonoids	Firmicutes/Bacteroidetes ratio	Dietary flavonoids modulate the Firmicutes/Bacteroidetes ratio and influence mucosal microbial composition.
	*Tenericutes*, *Deferibacteres*	Flavonoids alter microbial community structure within these phyla.
	*Prevotella* spp.	Flavonoids contribute to glucose metabolism and amino-acid fermentation by *Prevotella*.
Allicins	*Lactobacillus* spp., *Clostridia*	Allicin influences gut microbial composition and has been associated with blood-pressure–lowering effects.

This table has been developed and adapted based on the investigations of Beane et al. and Fu et al. (103, 104). It provides a summary of how deficiencies or variations in the availability of micronutrients, including vitamins and minerals, as well as plant-derived bioactive compounds influence both pathogenic and beneficial microorganisms. The reported effects include changes in inflammation, oxidative stress, intestinal barrier function, and overall microbial composition, underscoring the significance of these dietary components for host metabolic and immune health.

### 2.4 Influence of plant-based dietary patterns on the gut microbiota

#### 2.4.1 Vegetarian diet

A vegetarian diet is defined by the exclusion of specific foods and products derived from animals, either completely or partially. This dietary pattern encompasses several categories based on the inclusion of certain food items. One category is the ovo-lacto vegetarian diet, which allows for the consumption of both dairy products and eggs. Another variation is the ovo vegetarian diet, wherein individuals consume eggs but exclude dairy products. Conversely, the lacto vegetarian diet permits the intake of dairy products while excluding eggs. Additionally, there is the semi-vegetarian approach, which allows for the consumption of red meat, poultry, and fish no more than once a week. Finally, the pescatarian diet includes fish, as well as dairy products and eggs (105).

The adoption of a vegetarian diet has significant implications for the composition of gut microbiota, resulting in marked changes in the abundance of microorganisms at the genus, species, and strain levels. This dietary pattern is increasingly recognized as a viable nutritional intervention strategy, with the potential to regulate gut health and offer protection against inflammatory processes. Research indicates a greater abundance of certain bacterial genera, such as *Prevotella*, *Clostridium*, *Lactobacillus*, *Ruminococcus*, *Eubacterium rectale*, and *Faecalibacterium prausnitzii* among individuals following vegetarian diets, coupled with a reduced presence of *Bacteroides* and *Bifidobacterium* when compared to those adhering to omnivorous diets (105).

Furthermore, the recognition of vegetarianism as a healthy dietary approach is supported by emerging evidence of its physiological benefits. Vegetarians typically exhibit a lower body mass index, decreased serum cholesterol concentrations, lower prevalence rates of diabetes, and reduced blood pressure levels. A higher intake of plant-based foods, characterized by increased dietary fiber and antioxidants, has also been associated with a diminished risk of cardiometabolic conditions and other chronic diseases (106). This evidence underscores the potential of vegetarian diets to contribute positively to overall health outcomes.

#### 2.4.2 Vegan diet

The vegan diet is characterized by the exclusion of animal fats and proteins, which alters the amino acid profile and increases the intake of dietary fiber. These changes are associated with significant modifications in the gut microbiota, particularly via an increase in *Prevotella*, a microbial genus often linked to plant-based dietary patterns (107).

Vegan diets are associated with numerous health benefits, primarily due to their high concentrations of fiber, folic acid, vitamins C and E, potassium, magnesium, and a diverse array of phytochemicals. Furthermore, they typically contain a higher proportion of unsaturated fats compared to non-vegan diets. However, if not carefully planned, a vegan diet may lead to deficiencies in essential nutrients such as protein, iron, and vitamin B₁₂. Despite these potential limitations, recent studies indicate that the adoption of a vegan diet may facilitate improvements in metabolic syndrome, reduce the risk of cardiovascular disease, and provide clinical benefits in managing inflammatory conditions, including rheumatoid arthritis (108).

#### 2.4.3 Mediterranean diet

The Mediterranean diet is characterized by a high consumption of plant-based foods, including fruits, vegetables, legumes, whole grains, nuts, and extra virgin olive oil. It also promotes moderate intake of fish, lean meats, and dairy products, while restricting consumption of red and processed meats. This dietary pattern is associated with numerous health benefits, particularly in relation to cardiovascular health, metabolic function, and inflammation reduction, all of which contribute to an enhanced quality of life (109).

Emerging research indicates that adherence to a Mediterranean diet has a beneficial impact on gut microbiota. Specifically, it increases the abundance of short-chain fatty acid (SCFA)-producing bacteria, primarily *Faecalibacterium prausnitzii*, *Roseburia* spp., and *Bifidobacterium* spp. (110). These bacteria metabolize dietary fiber and polyphenols to produce butyrate and other SCFAs, which are essential for reinforcing the intestinal barrier and diminishing inflammatory markers. The presence of these microorganisms within a balanced gut microbiome plays a crucial role in improving lipid and glucose metabolism as well as modulating immune responses. Furthermore, a healthy microbiota is linked to a reduced incidence of chronic degenerative diseases, particularly those associated with cardiovascular health and metabolic syndrome (111).

#### 2.4.4 Flexitarian diet

The flexitarian diet, commonly referred to as the semi-vegetarian diet, emphasizes the predominant consumption of plant-based foods while permitting the occasional intake of animal products, such as lean meats, fish, seafood, eggs, and dairy (112). Current scientific reviews indicate that this dietary approach has a favorable impact on gut microbiota, attributable to its high dietary fiber content and the diversity of plant-based foods included.

Research demonstrates that the flexitarian diet fosters the proliferation of beneficial bacterial populations, akin to those observed in strictly vegetarian diets. Notably, there is an increased prevalence of Bacteroidetes, which are implicated in the fermentation of polysaccharides, as well as specific bacterial groups such as *Prevotella*, *Ruminococcus*, and *Roseburia*, which produce SCFAs (113). This distinct microbial composition is directly associated with elevated SCFA production, which confers various health benefits, including enhanced regulation of the immune system, diminished systemic inflammation, and improved cardiometabolic health. Furthermore, the inherent flexibility of the flexitarian diet facilitates long-term adherence and encourages a gradual transition toward healthier eating patterns. This characteristic may contribute to its effectiveness in promoting sustainable dietary change (114).

#### 2.4.5 Whole food plant-based diet

The whole food plant-based (WFPB) diet is characterized by a focus on the intake of natural and minimally processed plant-based foods, including fruits, vegetables, legumes, whole grains, seeds, and nuts. This dietary framework restricts or eliminates animal products and ultra-processed foods to the greatest extent possible. Research indicates that the WFPB diet promotes a beneficial microbial profile conducive to intestinal health. By favoring the growth of bacteria such as *Prevotella* and *Bacteroides*, this diet enhances the metabolism of complex polysaccharides and the production of health-promoting metabolites. Furthermore, it creates an optimal environment for the development of butyrate-producing bacteria, including *Faecalibacterium*, *Eubacterium rectale*, and *Roseburia* (115).

These microbial communities play a critical role in maintaining the integrity of the intestinal mucosa, decreasing intestinal permeability, modulating inflammatory responses, and supporting metabolic health. Consequently, adherence to a WFPB diet is associated with a more anti-inflammatory microbiome, greater microbial diversity, and a reduction in cardiometabolic risk markers (116).

#### 2.4.6 Comparison of plant-based dietary patterns

In comparing the dietary patterns previously discussed, it is evident that specific microorganisms confer substantial health benefits, including the reduction of systemic inflammation and the enhancement of cardiometabolic regulation. In particular, microorganisms associated with fiber fermentation and the production of short-chain fatty acids, specifically *Ruminococcus* spp., *Eubacterium* spp., *Faecalibacterium prausnitzii*, *Roseburia*, *Prevotella*, and *Bifidobacterium* spp., exhibit increased abundance with higher consumption of plant-based foods (117).

The composition and relative abundance of specific gut bacteria can vary significantly based on an individual’s dietary profile maintained over an extended period. For instance, research indicates that a whole-food, plant-based diet is associated with a microbiota that exhibits increased production of short-chain fatty acids compared to a flexitarian diet. Conversely, the Mediterranean diet, characterized by its high intake of foods rich in healthy fats and polyphenols, enhances specific metabolic pathways that are instrumental in modulating immune responses and regulating lipid metabolism (111, 113, 115).

Diets characterized by a higher intake of animal-based foods are associated with the proliferation of bacteria involved in protein fermentation, as well as an increase in metabolites that contribute to inflammation and elevate cardiometabolic risk (118).

Apparent inconsistencies across studies, such as variable reports on Bifidobacterium abundance in individuals following vegan diets, likely reflect differences in the specific composition of plant-based diets, including fiber type and intake, degree of food processing, geographic and cultural context, duration of dietary adherence, and methodological approaches used to assess the gut microbiota.

## 3 Conclusion

The gut microbiota is composed of a diverse array of microorganisms, predominantly bacteria from the phyla Firmicutes, Bacteroidota, Actinobacteria, and Verrucomicrobiota. These microorganisms engage in complex interactions, competing for nutrients and participating in metabolic processes crucial for human health. Through the utilization of available dietary substrates, these microbial communities ferment polysaccharides, synthesize essential vitamins, and produce bioactive metabolites such as short-chain fatty acids. These metabolites have been shown to exert beneficial effects on the immune system, support the intestinal barrier, promote energy metabolism, and modulate inflammation.

The composition, stability, and functionality of these microbial communities are heavily influenced by the type and availability of nutrients consumed by the host. A balanced and adequate intake of dietary fiber, monounsaturated and polyunsaturated fatty acids, plant-based proteins, polyphenols, vitamins, and minerals has been associated with the proliferation of beneficial genera, including *Bifidobacterium*, *Lactobacillus*, *Faecalibacterium*, and *Akkermansia*. These microorganisms play a key role in preserving the integrity of the intestinal mucosa, maintaining immune homeostasis, and mitigating the risk of metabolic, inflammatory, and chronic diseases.

In summary, the evidence supports the notion that plant-based dietary patterns represent an effective strategy for fostering a more diverse, stable, and functional gut microbiome, with significant implications for the overall health of the host.

### 3.1 Future outlook

While there is an increasing body of evidence supporting the role of plant-based diets in modulating gut microbiota, several limitations persist. Most available studies are cross-sectional or consist of short-term interventions, which restricts the ability to draw causal inferences and limits the interpretation of long-term effects. Future research should prioritize well-designed longitudinal studies that examine human dietary patterns over extended periods to enhance our understanding of the temporal relationships between diet, microbiota dynamics, and health outcomes. Furthermore, personalized nutrition approaches that account for individual variability in microbiota composition and metabolic responses require further investigation. Additionally, mechanistic studies focused on specific plant-derived compounds, such as distinct polyphenols and fibers, are essential for elucidating their direct interactions with gut microorganisms and the subsequent effects on host physiology.
